# Monitoring of Patients Who Presented With First Episode Psychosis, Under the Early Intervention Team, Against NICE Guidance for Psychosis and Schizophrenia

**DOI:** 10.1192/bjo.2022.455

**Published:** 2022-06-20

**Authors:** Rahul Khanna

**Affiliations:** Warrington and Halton Hospitals NHS Trust, Warrington, United Kingdom

## Abstract

**Aims:**

To assess the monitoring of patients who present with first episode psychosis (FEP), who are commenced on antipsychotics, under the early intervention team (EIT), in accordance with NICE guidelines. Patients diagnosed with FEP are usually treated with atypical antipsychotic's hence have a higher chance of developing metabolic syndrome, thus screening for physical health is imperative.

**Methods:**

This was a retrospective audit of patients with FEP, started on antipsychotics, under the EIT, between the 1st January 2020 to 31st December 2020. The date range allowed for a complete data set, as well as to assess the impact, if any, of COVID-19. A sample of 26 patients were identified by the EIT of which, once inclusion criteria was applied, 21 were audited.

Compliance was calculated on investigations being completed at every stage, as defined in the standards. For example: blood pressure had to be measured at 12 weeks and 1 year to be compliant with the standard. Data collection and analyses was completed using the IT system ‘Rio’ and Microsoft Excel.

**Results:**

There was an overall compliance rate of 51%. The results showed no patients had their weight/BMI monitored as per guidelines. Waist circumference was not measured in any patients. 43% met the monitoring standards for pulse and blood pressure. On further analysis, by 1 year 90% of patients had their pulse and blood pressure checked. Blood lipids were correctly monitored in 48% of cases, nevertheless when the results were broken down, 86% of patients had been monitored within a year. Prolactin monitoring occurred correctly in 52% of patients. Blood tests including full blood count, urea and electrolytes and liver function tests adhered to guidance greater than other parameters at, 86%, 86% and 90%, respectively. 48% of patients had plasma glucose/HbA1c monitored. An ECG at 1 year was obtained in 67% of the patients.

The compliance rate may have been lower than expected due to COVID-19 preventing in-person appointments, staff redeployment and disengagement from patients.

**Conclusion:**

Monitoring after antipsychotic medication has been commenced requires improvement. Within one year, monitoring was generally met well. However, the monitoring did not always meet the specific time frames provided by NICE, thus current systems need reviewing. Recommendations included disseminating results throughout the EIT, adding waist circumference as an option on the physical health assessment form and create ‘blood sample sets’. Re-audit will allow us to assess the results of interventions and the impact COVID-19 had on monitoring.

